# Decreased exercise capacity, strength, physical activity level and quality of life in adult patients with familial Mediterranean fever

**DOI:** 10.3906/sag-2011-98

**Published:** 2021-08-30

**Authors:** Nurten Gizem TORE, Fulden SARİ, Devrim Can SARAÇ, Selin BAYRAM, Hasan SATIŞ, Hazan KARADENİZ, Abdurrahman TUFAN, Deran OSKAY

**Affiliations:** 1 Department of Physiotherapy and Rehabilitation, Faculty of Health Sciences, Gazi University, Ankara Turkey; 2 Department of Internal Medical Sciences, Faculty of Medicine, Gazi University, Ankara Turkey

**Keywords:** Exercise capacity, familial Mediterranean fever, muscle strength, physical activity, quality of life

## Abstract

**Background:**

Familial Mediterranean fever (FMF) is a systemic autoinflammatory disease that causes recurrent attacks of fever, polyserositis, arthritis or skin eruptions, resulting in pain in the abdomen, muscles, joints and chest. All of these might lead to a reduction in exercise capacity, muscle strength, physical activity level (PAL) and quality of life (QoL). Therefore, assesment of these parameters are important. The aim of this study was to assess exercise capacity, muscle strength, PAL, and QoL in patients with FMF as compared to controls.

**Materials and methods:**

A total of 40 subjects with FMF and 36 healthy control subjects participated in the study. The 6-minute walk test (6MWT) was used to assess exercise capacity. Muscle strength measurements for shoulder flexors, extensors and abductors, hip flexors, extensors and abductors, knee flexors and extensors, and ankle dorsiflexors were evaluated by hand-held dynamometer. PAL was assessed using the International Physical Activity Questionnaire-Short Form (IPAQ-SF). QoL was investigated by Nottingham Health Profile (NHP).

**Results:**

Significant differences were found between patients and healthy subjects for 6MWT (p = 0.003), muscle strength of ankle dorsiflexors (p = 0.001), hip flexors (p = 0.047), extensors (p = 0.003) and abductors (p = 0.004), total scores of IPAQ-SF (p = 0.004), and pain (p < 0.001), physical mobility (p < 0.001) and energy level (p = 0.026) subscales of NHP. However, there were no significant differences between groups for the shoulder flexion (p = 0.089), extension (p = 0.440) and abduction (p = 0.232), hand grip strength (p = 0.160) , and knee flexion (p = 0.744) and extension (p = 0.155) muscle strength and emotional reaction (p = 0.088), sleep (p = 0.070) and social isolation (p = 0.086) subsets of NHP.

**Conclusion:**

Subjects with FMF demonstrated lower exercise capacity, muscle strength, PAL and QoL than healthy peers. Therefore, it is important to evaluate and improve these parameters in patients with FMF.

## 1. Introduction

Familial Mediterranean fever (FMF) is a common systemic autoinflammatory disease characterized by short-term recurrent attacks of fever, polyserositis and arthritis that results in pain in the abdomen, joints, muslces and chest [1,2]. It has been shown that the attacks are often associated with high levels of acute phase reactants, and last in 1 to 4 days [3,4]. In general, patients are symptom free in between attacks [4]. 

FMF is a lifelong disease; therefore, patients with FMF require daily colchicine utilization. However, this medication might lead to myopathy and decrease the muscle strength [5]. Furthermore, almost 10% of patients complain about muscle pain in the lower limb that arises after physical effort or prolonged standing [6]. Moreover, arthritis of FMF have an effect on joints during the attack period. It usually causes pain and swelling of a single and large joint of the lower limbs. Although this situation lasts between 1–3 days in most patients, it is rarely prolonged in some patients. Protracted arthritis can be seen 5% of FMF patients and mostly affect hips or knees [4]. In some cases, upper extremity involvement is also seen, yet, it is rare [7]. 

Chronic process of FMF may give rise to mood disturbances and disease related complications. The sypmtoms of rheumatic diseases are thought to cause an inactive lifestyle [8,9]. As stated in previous studies, physical inactivity and sedantary life may have detrimental effect on manifestations of rheumatic diseases by deteriorating muscle function, exercise capacity, and eventually quality of life (QoL) [10,11]. 

In the literature, it was indicated that young patients with FMF demonstrated lower exercise capacity, quadriceps muscle strength [11] and QoL [11,12] than healty controls. It was also remarked that QoL of adult patients with FMF is lower than healty controls [13,14]. In a study, it was found that FMF attacks considerably impair the physical activity of patients. However, in that study, physical activity levels of patients were not compared to healthy controls [15]. 

As mentioned earlier, arthralgia can be observed in upper extremities of patients with FMF, although not as often as in the lower limbs. Yet, there is no study in the literature that examines the upper limb muscles’ strength and hand grip strength of patients with FMF. Besides, when the studies were investigated, it was determined that only quadriceps muscle strength was examined for the lower extremity [11]. However, only quadriceps muscle do not reflect the strength of the lower limb muscles. Muscles such as ankle dorsiflexors, hip flexors, hip abductors, and so on are also important for gait [16], and these muscles might affect the physical activity level [17] and aerobic capacity [18] of patients. 

Only one study in the literature evaluated quadriceps muscle strength and aerobic capacity in children with FMF. According to the results of this study, it was found that children with FMF had lower quadriceps muscle strength and aerobic capacity compared to healthy controls [11]. There is a lack of research in the literature on upper and lower extremity muscle strength, exercise capacity, and physical activity level in adults with FMF. It is important to evaluate lower and upper extremity muscle strength. Arthritis, which occurs with attacks and sometimes prolongs, might affect the joints in the lower and upper extremities. As a result, patients may experience physical inactivity. Increased physical inactivity might cause a decrease in aerobic capacity. All of these might lead to worsening of the QoL in patients with FMF. Therefore, the above-mentioned parameters should be evaluated. The aim of this study was to investigate exercise capacity, strength, physical activity level and QoL of patients with FMF as compared to healthy controls.

## 2. Materials and methods

### 2.1. Study design

The research was designed as a case-control study. The ethical approval was obtained by the local ethical committee. All participants were informed about the study. They signed a written consent, and the research was conducted in compliance with Declaration of Helsinki. 

### 2.2. Participants

Forty participants who diagnosed with FMF (male/female: 12/28) and fulfilling the Tel Hashomer criteria, presented to the rheumatology department of the university hospital for routine control by a rheumatologist and were found to be in remission by their physician were included in the study. Patients who were older than 65 years old or younger than 18 years old, in the attack period during the evaluation, had any orthopedic problem such as congenital problems, amputation, etc. in their lower extremities that prevents them from walking in order to perform 6MWT, diagnosed with any rheumatic disease in addition to FMF, diagnosed with hypertension, and who were illiterate were excluded from the study. Since, the questionnaires used in the evaluation take into account the last 1 week, patients who had an attack in the last 2 weeks were also excluded from the study. The healthy controls were matched by age and sex with patients with FMF. Healthy controls were selected randomly. Thirty-six adults, 18 to 65 years old, with no known medical conditions were included in the study as control group. The healthy controls were matched for sex and age. Demographic data containing age, dominant hand, body mass index (BMI), duration of the disease and family history were recorded. In addition, the participants were questioned about whether they have exercise habits, complain about myalgia in their lower extremities. Moreover, the number of attacks in the last 3 months, the drug they used and the daily dose of this drug were also recorded. 

### 2.3. Measures

Exercise capacity: The 6-minute walk test (6MWT) was used to assess exercise capacity of the participants since, in general, daily living activities are performed at submaximal levels of effort. Thus, it was recommended that submaximal functional tests demonstrate better reflection of physical ability. Additionally, 6MWT is well tolerated by patients, easy and has been widely used as an outcome measure to assess exercise capacity of patients with chronic disease [19]. Baseline dyspnea, overall fatigue and leg fatigue questioned according to the Borg scale and vital signs (heart rate, blood pressure, oxygen saturation and respiration rate) of participants were recorded before the 6MWT. Due to the learning effect, as stated in the guidelines, the participants performed this test on the 30-m track, 2 times with an interval of 30 min. Before the tests, participants were given the same verbal instructions. All information about the test was given to the participants before starting the test, then, they asked to walk the maximum distance that they can do without jogging or running in a 6-min period. They were allowed to slow down, to stop or to rest if necessary, and continue to walk as immediate as possible. The researchers kept the participants informed about the remaining time, and ensured encouragements after each minute during the test. After the test was completed and recovery time (1 min after the end of the test), the data recorded before the test were reevaluated [20]. The total walking distance was recorded in meters.

Strength*: *Hand-held dynamometer (Lafayette Manual Muscle Tester, Model 01,163, Lafayette Instrument Company, Lafayette, IN, USA) was used to evaluate muscle strength of the participants. Shoulder flexor, extensor and abductor, hip flexor, extensor and abductor, knee flexor and extensor and ankle dorsiflexor muscles’ strengths were assessed by the same researcher. Measurement for all muscles was performed as described in previous studies [21,22]. Only dominant extremities were evaluated. The test was repeated 3 times with an interval of 30 s, and the avarage score was recorded. 

Grip strength was evaluated using a hand-held dynamometer. (Jamar, Sammons Preston, Bolingbrook, IL, UK). This assessment was conducted with the participant in an upright position, sitting on a chair with the back supported, the arm of the participant was in adduction and the elbow in flexion of 90° [23]. Each assessment was repeated 3 times for dominant hand. Then, the avarage score of the 3 repetead measurements was recorded. 

Physical activity level: Turkish version of the International Physical Activity Questionnaire-Short Form (IPAQ-SF) was used to assess physical activity level of participants. This questionnaire comprise of 7 questions asking individuals to remember the physical activity of the previous week. It inquires about the total of time that participants spent sitting, walking or doing moderate (e.g., doubles tennis) or vigorous activities (e.g., digging aerobics). For IPAQ-SF, physical activities fall into 4 different domains: activities of leisure time, gardening and domestic activities, work-related activities, and transportation. As a result of the calculation of total score, the physical activity level can be divided into 3 groups as low, medium and high. A higher total score from the questionnaire indicates greater physical activity [24]. 

Health related quality of life (HRQoL): The Nottingham Health Profile (NHP) was used to evaluate HRQoL of the participants. NHP was developed to assess HRQoL in different perspectives. It is a general self-evaluation of subjective status of health in several areas and can be completed in a short time. It comprises of 2 parts. The first part of NHP consist of questions that inquires about currently experienced problems, which are related to social, psychological and physical functioning associated with health status. It focuses on 6 dimensions and includes 38 questions, which are related to pain, vital energy, sleep disorders, physical fitness, emotional reaction and social isolation. The second part of NHP contains 7 questions that referred to housework, paid work, social, family and sexual life, hobbies and interests, and the use of leisure time. Higher score indicates greater severity of health problems [25]. 

### 2.4. Statistical analysis

Data analysis were conducted using Statistical Package for the Social Sciences (SPSS Inc. Version 21; IBM, Raleigh, NC, ABD) for Windows. Normality distribution of data was tested by using Shapiro–Wilk test. Descriptive data were expressed as means ± standart deviation (sd) where appropriate. To compare two groups, Student t test (age, sex, BMI (body mass index), dominant hand, exercise habit, muscle strength, grip strength, 6MWT, IPAQ-SF and NHP) was utilised. The p values were accepted significant at <0.05. 

## 3. Results

Forty patients with FMF and 36 healthy individuals were included in this study. The mean ages of patients with FMF and healthy control group were 34.97 ± 11.6 years and 30.88 ± 8.88 years, respectively. The mean BMI was 25.48 ± 4.77 for the patients and 23.88 ± 3.84 for the healthy subjects. There were no significant differences between FMF and the control group with regards to gender (p = 0.959), age (p = 0.087) and BMI (p = 0.111). Other demographic characteristics of participants are listed in Table 1.

**Table 1:** Characteristics of the participants.

Characteristic	ControlN = 36	FMFN = 40	p
Age, years	30.88 ± 8.88	34.97 ± 11.6	0.087
Sex, female/male	27/9	28/12	0.959
BMI, kg/m2	23.88 ± 3.84	25.48 ± 4.77	0.111
Dominant hand, right/left	35/1	39/1	0.941
Disease duration, years	n.a.	10.02 ± 7.26	n.a.
Colchicine dose, mg, median ± SD	n.a.	1.52 ± 0.55	n.a.
Myalgia, yes/no	n.a.	23/17	n.a.
Exercise habit, yes/no	11/25	6/34	0.112
Number of attacks in last 3 months, median (min-max)	n.a.	1.5 (0-7)	n.a.

FMF: familial Mediterranean fever, cm: centimeter, kg: kilogram, m: meter, mg: milligram, n.a.: not applicable

The mean grip strength value of patients and control group were 28.16 ± 12.11 and 31.84 ± 10.28, respectively. There was not any significant difference between 2 groups in terms of grip strength (p = 0.160). Half of the healthy control group were low, and half were moderately physically active. Of those with FMF, 29 were low and 11 were moderately physically active. There was a statistically significant difference between 2 groups in regards to activity categories of IPAQ (p = 0.044) (Table 2). According to the results of this study, when muscle strength is taken into account, the mean muscle strength of hip flexors was 221.31 ± 66.05 in the healthy control group and 192.50 ± 58.49 in the FMF group, the mean muscle strength of the hip extensors was 241.95 ± 95.82 in the healthy control group and 184.08 ± 65.11 in FMF group. The mean muscle strength of the hip abductors was 265.95 ± 78.60 in the healthy control group and 212.24 ± 76.56 in the FMF group, the mean muscle strength of ankle dorsiflexors was 251.97 ± 85.08 in the healthy control group and 192.65 ± 63.14 in the FMF group. Considering the mean 6MWT results, it was 633.61 ± 57.97 in the healthy control group and 565.65 ± 72.31 in FMF group. Considering the mean IPAQ (MET) values, it was 911.95 ± 771.21 in the healthy control group and 463.61 ± 482.69 in FMF group. The rest of muscle strength measurements’of participants are listed in Table 2. Significant differences were found between patients and healthy subjects for muscle strength of ankle dorsiflexors (p = 0.001), hip flexors (p = 0.047), extensors (p = 0.003) and abductors (p = 0.004), 6MWT (p = 0.003), and total scores of IPAQ (p = 0.004). These results indicate that patients with FMF have lower muscle strength, exercise capacity and physical activity level than healthy controls. According to the results of NHP subscales, the mean results for pain subscale was 6.63 ± 15.46 in the healthy control group and 32.67 ± 27.34 in FMF group, the mean results for physical mobility subscale was 3.00 ± 6.12 in the healthy control group and 16.73 ± 14.72 in FMF group, and the mean results for the energy level subscale was 23.53 ± 33.60 for the healthy control group and 42.96 ± 40.73 for FMF group. The remaining results of subscales of NHP are listed in Table 3. Significant differences were found between patients and healthy subjects for pain (p < 0.001), physical mobility (p < 0.001) and energy level (p = 0.026) subscales of NHP, which remarks lower QoL of patients with FMF than healthy controls.

**Table 2:** Muscle strength, exercise capacity characteristics and physical activity level of participants.

Assessment	ControlN = 36	FMFN = 40	p
Shoulder flexors, N	173.78 ± 61.18	150.16 ± 58.4	0.089
Shoulder extensors, N	150.23 ± 49.49	140.41 ± 59.56	0.440
Shoulder abductors, N	161.78 ± 49.12	146.61 ± 59.40	0.232
Hip flexors, N	221.31 ± 66.05	192.50 ± 58.49	0.047
Hip extensors, N	241.95 ± 95.82	184.08 ± 65.11	0.003
Hip abductors, N	265.95 ± 78.60	212.24 ± 76.56	0.004
Knee flexors, N	144.36 ± 45.75	140.85 ± 47.37	0.744
Knee extensors, N	221.46 ± 62.54	199.70 ± 68.82	0.155
Ankle dorsiflexors, N	251.97 ± 85.08	192.65 ± 63.14	0.001
Grip strength, N	31.84 ± 10.28	28.16 ± 12.11	0.160
6MWT, meter	633.61 ± 57.97	565.65 ± 72.31	0.003
IPAQ, MET	911.95 ± 771.21	463.61 ± 482.69	0.004
IPAQ, categoryLowModerate	1818	2911	0.044

N: Newton, 6MWT: 6-minute walk test, MET: metabolic equivalent, IPAQ: International Physical Activity Questionnaire,

**Table 3:** Quality of life characteristics of participants.

Assessment	ControlN = 36	FMFN = 40	p
NHP Pain	6.63 ± 15.46	32.67 ± 27.34	<0.001
NHP Emotional reactions	11.85 ± 17.50	20.25 ± 24.51	0.088
NHP Sleep	17.57 ± 26.11	19.90 ± 27.08	0.070
NHP Social isolation	6.79 ± 13.78	13.77 ± 20.17	0.086
NHP Physical mobility	3.00 ± 6.12	16.73 ± 14.72	<0.001
NHP Energy level	23.53 ± 33.60	42.96 ± 40.73	0.026

NHP: Nottingham Health Profile.

## 4. Discussion

According to the results of this study, it was found that hip muscles’ strengths, ankle dorsiflexion muscle strength, functional capacity, physical activity level, and QoL were significantly lower in patients with FMF than healthy subjects. To the best of our knowledge, this is the first research investigating the exercise capacity, strength, and physical activity level in adult patients with FMF.

It is known that chronic diseases give rise to both psychological and physical symptoms, and these symptoms lead to difficulties in daily living activities and QoL [26]. Increasing the QoL in individuals with chronic diseases is one of the most important goals of treatment. HRQoL is a multidimensional concept, which focuses on subjective perception of emotional, social, and physical functioning [27]. Therefore, evaluation of HRQoL is important to understand the effects of disease on patients’ life. HRQoL can be assessed easily by patient reported instruments. Thus, NHP was used in this study to evaluate HRQoL of adult patients with FMF. As a result of this study, it was observed that scores of pain, physical mobility, and energy level sections were significantly lower in patients with FMF than healthy controls. Prior studies indicated that QoL is considerably impaired in children and young patients with FMF than healty subjects [11,12]. Furthermore, the same results were found in studies examining the QoL of adult FMF patients [13,14,28]. The fact that the disease progresses with attacks, the emergence of symptoms during the attack period, etc. affects the QOL, as seen in other studies, was an expected result. Our findings regarding HRQoL are compatible with the previous studies.

As mentioned earlier, musculoskeletal symptoms are common in patients with FMF. Arthritis, artralgia, and myalgia might affect mostly hips or knees [4]. Since the attacks of these patients are painful and physical exertion causes attacks, patients turn to an inactive lifestyle [15]. This situation cause limitations in physical performance in daily living activities. For this reason, patients with FMF might have more problems in performing daily living activities due to lower physical activity level and exercise capacity than healthy individuals. In this study, the mean distance for 6MWT covered by patients was statistically lower than healthy controls. In a previous study, it was found that the exercise capacity of children patients with FMF was lower than the healthy control group [11]. Our results regarding exercise capacity are compatible with the literature. The reason for this result might be, as stated in Babaoglu et al.’s study, patients are physically inactive during the attack period [15]. Moreover, given EULAR (European League Against Rheumatism) recommendations, patients are informed by their physicians that physical stress triggers FMF attacks [29]. All of these result in a decrease in exercise capacity.

It has been proven in previous studies that the symptoms seen in rheumatological diseases cause an inactive lifestyle [30,31]. We thought that the symptoms of the musculoskeletal system mentioned above, occurring during the attack period and sometimes prolonged in patients with FMF would decrease the level of physical activity by causing an inactive lifestyle as in other rheumatological diseases. Therefore, the physical activity level of these patients was evaluated. In this study, physical activity level of patients with FMF was found lower than healthy controls. In a study evaluating the physical activity of patients with FMF, it was determined that the number of steps of these patients decreased statistically significant in the attack period compared to the number of steps in the non-attack period [15]. However, there is no study comparing the physical activity level of healthy controls and patients with FMF.

Joint pain is the third most common clinical finding of FMF after fever and abdominal pain. An articular FMF attack generally presents as monoarthritis, and it often has an effect on the large joints of lower limbs. It was indicated that swelling of joints and presence of joint pain often lead to decreases in muscle mass and strength in patients with inflammatory arthritis. Moreover, myalgia is one of the findings of FMF. It occurs in about 10% of patients and it frequently affects calf and thigh muscles. Myalgia can be improved by rest [32]. This, in turn, might lead to physical inactivity of patients, resulting in decreased muscle strength indirectly. Furthermore, in musculoskeletal diseases, muscle strength can be affected by disease duration, drug-induced myopathy, and joint status [8]. Although it is not as frequent as the lower extremity, it has been reported in the literature that the musculoskeletal system is also affected in the upper extremity [7]. For this reason, we investigated the strength of shoulder, hip, knee and ankle muscles, which are used in daily living activities. In addition, hand grip strength was also evaluated. According to the muscle strength measurement results, a significant difference was found between healthy individuals and patients with FMF for the ankle dorsiflexor, hip flexor, extensor and abductor muscles. Yet, there was no significant difference between shoulder flexors, extensors and abductors, knee flexors and extensors, and hand grip strength between healthy individuals and patients with FMF. Upper limb muscles’ strength and hand grip strength were lower in FMF patients compared to healthy subjects; however, no statistically significant difference was found. Our findings regarding upper extremity muscle strength and hand grip strength are compatible with prior studies. Muscle strength of hip muscles, knee flexors and ankle dorsiflexors has not been evaluated in previous studies. A study evaluating knee extensors found that young patients with FMF had a lower knee extensor strength than healthy subjects [11]. Yet, in our study, although the quadriceps muscle strength of FMF patients was lower compared to the healthy subjects, no statistically significant difference was found between two groups. The previous study was conducted with 100 children with FMF and 55 healthy subjects [11]. The reason why there was no statistically significant difference in knee muscle strength in this study might be the lower number of participants compared to the previously mentioned study. It has been stated in the literature that all lower extremity muscles affect walking levels and physical activity level; however, hip abductor muscles and ankle dorsi flexor muscles play an important role in walking parameters [33]. Considering this information, the weakness of the hip and ankle muscles may explain that the patients with FMF demonstrate lower physical activity levels and, thus, exercise capacity compared to healthy individuals. 

The lack of correlation analysis for the parameters evaluated is a limitation of this study. These analyzes are recommended for future studies.

All in all, patients with FMF demonstrated lower physical activity level, exercise capacity, QoL, muscle strength of ankle dorsiflexors, hip flexors, extensors and abductors than healthy controls. Therefore, evaluation of these parameters in patients with FMF is of great importance in terms of elimination of muscle weakness, increasing physical activity level and aerobic capacity, and improving the QoL.

## Informed consent

Ethics committee approval was obtained for this study and all participants signed an informed consent form.
